# Strategies to Optimize Microalgae Conversion to Biogas: Co-Digestion, Pretreatment and Hydraulic Retention Time

**DOI:** 10.3390/molecules23092096

**Published:** 2018-08-21

**Authors:** Maria Solé-Bundó, Humbert Salvadó, Fabiana Passos, Marianna Garfí, Ivet Ferrer

**Affiliations:** 1 GEMMA—Group of Environmental Engineering and Microbiology, Department of Civil and Environmental Engineering, Universitat Politècnica de Catalunya·BarcelonaTech, c/Jordi Girona 1-3, Building D1, E-08034 Barcelona, Spain; maria.sole-bundo@upc.edu (M.S.-B.); marianna.garfi@upc.edu (M.G.); 2 Department of Evolutionary Biology, Ecology and Environmental Sciences, Faculty of Biology, Universitat de Barcelona, Av. Diagonal 643, 08007 Barcelona, Spain; hsalvado@ub.edu; 3 Department of Sanitary and Environmental Engineering, Federal University of Minas Gerais, Antonio Carlos Avenue 6627, 31270-090 Belo Horizonte, Brazil; fabiana@desa.ufmg.br

**Keywords:** anaerobic digestion, bioenergy, co-digestion, hydraulic retention time, microalgal biomass, primary sludge, thermal pretreatment

## Abstract

This study aims at optimizing the anaerobic digestion (AD) of biomass in microalgal-based wastewater treatment systems. It comprises the co-digestion of microalgae with primary sludge, the thermal pretreatment (75 °C for 10 h) of microalgae and the role of the hydraulic retention time (HRT) in anaerobic digesters. Initially, a batch test comparing different microalgae (untreated and pretreated) and primary sludge proportions showed how the co-digestion improved the AD kinetics. The highest methane yield was observed by adding 75% of primary sludge to pretreated microalgae (339 mL CH_4_/g VS). This condition was then investigated in mesophilic lab-scale reactors. The average methane yield was 0.46 L CH_4_/g VS, which represented a 2.9-fold increase compared to pretreated microalgae mono-digestion. Conversely, microalgae showed a low methane yield despite the thermal pretreatment (0.16 L CH_4_/g VS). Indeed, microscopic analysis confirmed the presence of microalgae species with resistant cell walls (i.e., *Stigioclonium* sp. and diatoms). In order to improve their anaerobic biodegradability, the HRT was increased from 20 to 30 days, which led to a 50% methane yield increase. Overall, microalgae AD was substantially improved by the co-digestion with primary sludge, even without pretreatment, and increasing the HRT enhanced the AD of microalgae with resistant cell walls.

## 1. Introduction

Algal biofuels call for low-cost technologies to be competitive with fossil fuels. In this context, microalgae cultivation in wastewater reduces freshwater and nutrient consumption while providing sanitation. Microalgal-based wastewater treatment systems consist of open ponds (e.g., high rate algal ponds (HRAPs)) capable of removing organic matter without aeration in the biological reactor, as with conventional activated sludge systems. Indeed, heterotrophic bacteria use the oxygen released through microalgae photosynthesis. The biomass grown in the ponds is then harvested to obtain a clarified effluent. Harvested biomass can be valorized as an organic fertilizer [1] or to produce bioenergy, with anaerobic digestion (AD) being the most straightforward technology for this purpose [2,3].

However, microalgae AD is limited by their resistant cell wall, which hampers the conversion into methane [4]. Thus, the application of pretreatment methods to damage or weaken the microalgae cell wall increases the bioavailability of intracellular contents to anaerobic microorganisms [5,6]. Even so, some pretreatments might result in higher costs (e.g., chemicals or biological products) or energy requirements (e.g., thermal or mechanical techniques) than the benefits obtained by implementing the pretreatment step (energy gain). This is a relevant aspect when choosing the most appropriate pretreatment for each substrate [7]. In this sense, microalgae thermal pretreatment at low temperature (<100 °C) has shown a promising energy balance [8].

In addition, the high nitrogen content (i.e., low C/N ratio) of microalgae can lead to methanogen inhibition due to ammonia toxicity during the AD process [9,10]. To overcome this issue, possible solutions include the reduction of protein levels in microalgae biomass by culturing them in low nitrogen media or the use of ammonia-tolerant anaerobic inoculum [11,12]. More commonly, the co-digestion (i.e., the simultaneous digestion of two or more substrates) of microalgae with other carbon-rich biomass has been proposed to reduce the ammonia concentration levels in the reactors while increasing the organic loading rate (OLR) [6,13]. In such a case, co-substrates obtained near or at the same treatment plant are preferred to avoid transport costs [14]. This strategy could be easily implemented in microalgal-based wastewater treatment plants (WWTPs), where harvested microalgal biomass could be co-digested with primary sludge from primary settlers. Indeed, primary sludge is more readily digestible and has less protein content than microalgae [15], so it could enhance microalgae biodegradability while increasing the OLR. To the best of our knowledge, only a few studies have evaluated the co-digestion of microalgae with primary sludge and always in batch tests [15,16]. Given that some benefits were pointed out (e.g., methane yield increase), these results should be validated better in continuous reactors.

The aim of this study is to optimize the AD process in WWTPs based on HRAP. Thus, the co-digestion of primary sludge from primary settlers and harvested microalgal biomass from HRAP (hereafter called microalgae) was investigated in both batch and continuous reactors. Moreover, a thermal pretreatment at 75 °C for 10 h was applied to microalgae, and the HRT of anaerobic digesters was increased to evaluate their effect on the microalgae methane yield. Microscopic analyses were used to help in understanding how microalgae were degraded during the pretreatment and AD process. Finally, an energy assessment of each studied scenario was calculated to attest the viability of full-scale application.

## 2. Results

The co-digestion of microalgae and primary sludge at different proportions was initially studied by means of biochemical methane potential (BMP) tests (Section 2.1.1). Subsequently, two continuous lab-scale anaerobic reactors were run in parallel (Table 1). During the first period, the co-digestion of pretreated microalgae with primary sludge was investigated (Section 2.1.2). During the second one, microalgae mono-digestion (with and without pretreatment) at longer HRT was compared (Section 2.2.1), including a microscopic analysis (Section 2.2.2).

### 2.1. Improving Microalgae Anaerobic Digestion by Co-Digestion with Primary Sludge and Thermal Pretreatment

#### 2.1.1. Anaerobic Co-Digestion of Microalgae and Primary Sludge in Batch Tests

The co-digestion of microalgae with primary sludge was evaluated at different proportions (25%, 50% and 75% of microalgae, on a volatile solids (VS) basis) (Table 2). Additionally, in some trials, microalgae were pretreated at 75 °C for 10 h in order to solubilize the biomass and enhance the anaerobic digestion rate and extent [8]. Indeed, the microalgae methane yield was increased by 62% (from 90 to 146 mL CH_4_/g VS) and the first-order kinetics constant (*k*) by 128% (from 0.07 to 0.16 day^−1^) after the pretreatment (Table 2). However, primary sludge showed the highest methane yield (380 mL CH_4_/g VS) and faster kinetics (*k* = 0.24 day^−1^) as compared to untreated and pretreated microalgae. This is due to the nature of primary sludge, which is more readily digestible than microalgae.

However, the co-digestion of microalgae with primary sludge substantially improved the anaerobic digestion kinetics (*k* = 0.25–0.28 day^−1^) as compared to mono-digestion trials. Also, when comparing the experimental values of kinetics from co-digestion trials with those values calculated from the theoretical curves obtained as the sum of mono-digestion experimental values (Table 2), the experimental *k* value was always higher than the theoretical one. This means that mixing both substrates accelerated the AD process, as already observed in other cases [17,18]. This could contribute to reducing costs by decreasing the digesters’ hydraulic retention time (HRT) and thus their volume. Still regarding the kinetics, no differences were observed between pretreated and untreated trials, since microalgae and primary sludge co-digestion without pretreatment already improved by far the anaerobic digestion rate. On the contrary, the pretreatment itself had already accelerated the kinetics of the process, so the effects of the co-digestion results were less discernible than for untreated substrates [19,20].

**Figure 1:**
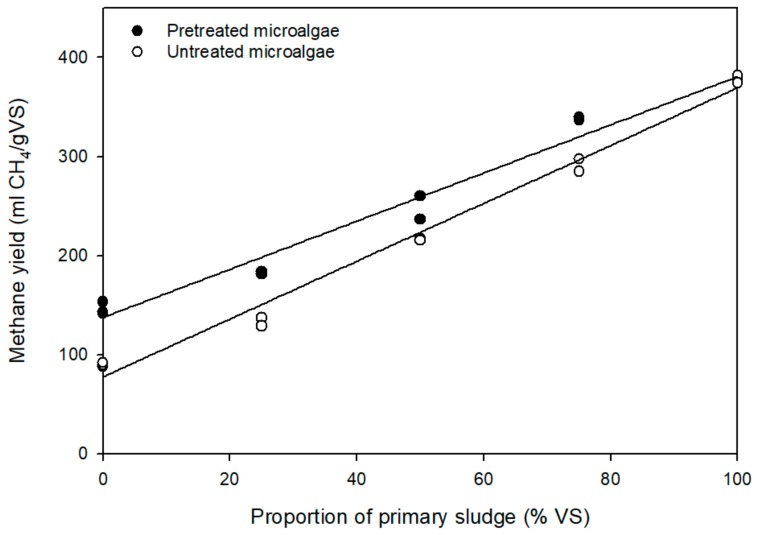
Correlation between the methane yield and the primary sludge proportion added to untreated and pretreated microalgae.

Otherwise, the higher the proportion of primary sludge, the higher the methane yield (Figure 1), with 339 mL CH_4_/g VS being the highest methane yield achieved with the co-digestion of 75% primary sludge and 25% pretreated microalgae. These findings suggest that there was no synergic effect with respect to the ultimate methane production when co-digesting both substrates.

#### 2.1.2. Anaerobic Co-Digestion of Microalgae and Primary Sludge in Lab-Scale Reactors

**Figure 2:**
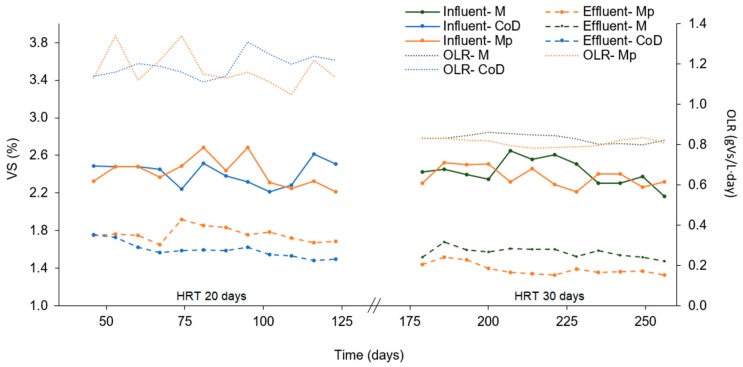
Influent and effluent volatile solids of untreated microalgae (M), thermally pretreated microalgae (Mp) and in co-digestion with primary sludge (CoD) for the studied periods: Period I at a HRT of 20 days and Period II at a HRT of 30 days.

The best co-digestion condition (25–75% VS of thermally pretreated microalgae and primary sludge) from BMP tests was thereafter compared to the mono-digestion of thermally pretreated microalgae in lab-scale reactors (Table 3, Figure 2). During the whole experimental period, both reactors were operated with an OLR of around 1.2 g VS/(L·day), given the concentration of VS in microalgae harvested and thickened by gravity (around 4% TS and 2.5% VS) and the HRT (20 days).

In the co-digestion reactor, the average methane yield was 0.46 L CH_4_/g VS, which represented a 2.9-fold increase as compared to pretreated microalgae mono-digestion (0.16 L CH_4_/g VS). In addition, the methane production rate increased from 0.20 to 0.53 L CH_4_/(L·day). Despite this important increase in methane yield and methane production rate, the average VS removal was not much different (34.3% for co-digestion vs. 27.9% for mono-digestion). A possible reason for this is that primary sludge had a higher lipid content than microalgae, which are mainly composed of proteins. Indeed, our previous study quantified the content of lipids (45% and 24% VS) and proteins (29% and 58% VS) for primary sludge and microalgae, respectively [13]. Comparing the methane potential of both macromolecules, lipids can achieve 1.014 L CH_4_/g VS and proteins only 0.851 L CH_4_/g VS [21]. Therefore, the conversion potential of primary sludge to methane is higher than microalgae, as already observed in the BMP tests. The methane yield of the co-digestion reactor was higher than that obtained co-digesting sewage sludge with *Spirulina maxima* (50% VS each) at 20 days of HRT (0.36 L CH_4_/g VS) [22], and similar to that obtained co-digesting *Scenedesmus* sp. or native microalgal biomass (25% VS) with sewage sludge (75% VS) at 15 days of HRT (0.39 and 0.51 L CH_4_/g VS, respectively) [23].

Concerning the stability of digesters, pH values were stable during the whole period, ranging from 7.35 to 7.55 (Table 3). Regarding the ammonium concentration, the highest value was observed in the mono-digestion reactor with pretreated microalgae (1.1 g N-NH_4_/L) due to a higher protein release during the AD process. This value is close to the threshold which resulted in AD inhibition [24]. Therefore, if reactors had been operated at higher OLRs, the inhibition of ammonia toxicity may have occurred. Conversely, co-digestion with primary sludge reduced the ammonium concentration in the digester to 0.6 g N-NH_4_/L. In this case, the OLR could have been increased without approaching the ammonia inhibition threshold. VFA concentrations were also very low in both reactors (Table 3) Finally, an important aspect for the digestate management and final disposal is its dewaterability. While the digestate from thermally pretreated microalgae digestion presented a poor dewaterability (CST value of 982 s), the results were consistently improved by the co-digestion with primary sludge (CST value of 290 s). In this sense, the co-digestion substantially improved the effluent dewaterability since primary sludge has less affinity for water than microalgae.

### 2.2. Effect of the Thermal Pretreatment on Microalgae Anaerobic Digestion

#### 2.2.1. Anaerobic Digestion of Thermally Pretreated Microalgae in Lab-Scale Reactors

As previously discussed, microalgae showed a low methane yield despite the thermal pretreatment (0.16 L CH_4_/g VS). In order to improve their anaerobic biodegradability, the digester HRT was increased from 20 to 30 days. In parallel, another digester with untreated microalgae was operated as control. During this period, the methane production rate of pretreated microalgae increased by 58% (from 0.12 to 0.19 L CH_4_/(L·day)) and the methane yield by 71% (from 0.14 to 0.24 L CH_4_/g VS) as compared to control (Table 3). Accordingly, the VS removal also increased from 36.2 to 39.5% (Table 3).

Regarding the ammonium concentration, it was higher in the pretreated reactor digestate than in the control (0.8 g N-NH_4_/L vs. 0.7 g N-NH_4_/L), suggesting a higher protein solubilization in the case of pretreatment. However, as a result of increasing the HRT, the OLR decreased from 1.2 to 0.8 g VS/(L·day). Consequently, the N-NH_4_ concentration in the reactor was reduced in comparison with the previous period at 20 days of HRT (0.8 vs. 1.1 g N-NH_4_/L).

The methane yield increase observed in this study is in agreement with the results obtained by Passos and Ferrer [8], who reported an increase of 70% after applying a thermal pretreatment at 95 °C for 10 h to similar microalgae species. However, different conclusions regarding the effect of the thermal pretreatment on microalgae can be found in the literature. For instance, no significant effect was observed after a pretreatment at 70 °C for 3 h to *Scenedesmus* sp., but the same pretreatment at 90 °C enhanced the anaerobic biodegradability of *Scenedesmus* sp. from 22 to 48% in BMP tests [25]. Other authors found no influence of the thermal pretreatment, but did find an effect of the thermochemical pretreatment, which increased methane yield by 40% in some microalgae species [26]. Indeed, the effect of the thermal pretreatment highly depends on the microalgae species and the conditions applied, and so a pilot-scale evaluation of the pretreatment performance is required before scaling-up.

In terms of digestate dewaterability, both the untreated and thermally pretreated microalgae showed a poor dewaterability, with higher CST values (795 and 919 s, respectively) than the co-digestion reactor (290 s).

#### 2.2.2. Microscopic Analysis

**Figure 3:**
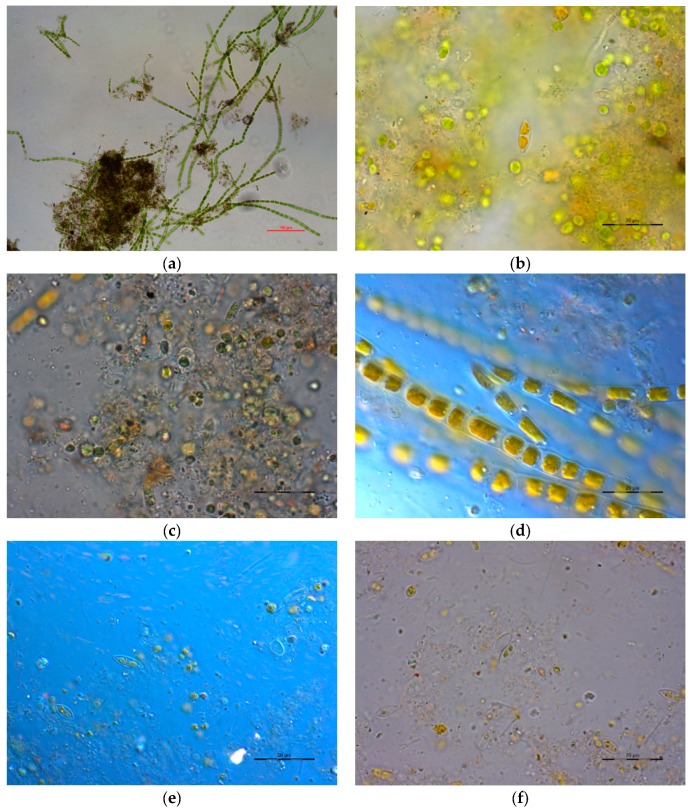
Microscopic images of microalgae before (**a**,**b**) and after (**c**,**d**) the thermal pretreatment, along with the digestates from untreated microalgae AD (**e**) and thermally pretreated microalgae AD (**f**) at a HRT of 30 days.

Microalgae were periodically characterized by optical microscopy over the whole experimental period. Qualitative results showed how microalgal biomass was flocculated. The main green microalgae species belonged to the genus *Chlorella* and *Stigeoclonium*, along with diatoms (Figure 3a,b). These microalgae species remained predominant during the whole period, although the relative abundance varied over time, which is common in open ponds treating wastewater [27].

After the thermal pretreatment, microalgae clearly appeared to be less pigmented than fresh microalgae and most of the cells were dead (Figure 3c,d). Also, in the pretreated sample, a higher amount of amorphous material was found because of organic matter release. However, most of the cell walls were found unbroken. This was especially the case for diatoms (Figure 3c) and *Stigeoclonium* sp. (Figure 3d), which presented a higher resistance to the pretreatment. Indeed, other authors concluded that the thermal pretreatment was not able to break microalgae cell walls but it did damage or weaken them [28,29].

To further evaluate the effect of the thermal pretreatment on microalgae AD, microscopic images from the digestate of pretreated microalgae (Figure 3f) were compared to those from the digestate of untreated microalgae (Figure 3e). In this manner, it was possible to elucidate whether pretreated cells were more accessible to methanogens, even if cell walls were not lysed after the pretreatment step. A higher amount of particulate substances was observed in the untreated microalgae digestate (Figure 3e), although entire microalgae cells were found in both digestates even after 30 days of digestion.

**Figure 4:**
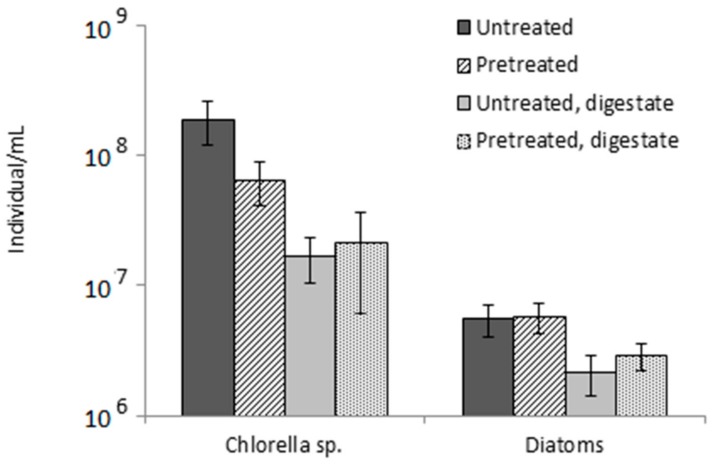
*Chlorella* sp. and diatoms counting in the influents (untreated; pretreated) and effluents (untreated digestate; pretreated digestate) during Period II. Mean values and standard deviation are represented.

Next, a quantitative analysis was conducted by counting the two most abundant microalgae species, *Chlorella* sp. and diatoms, in the influent and effluent (Figure 4). This analysis confirmed the qualitative results. While the number of *Chlorella* sp. individuals was reduced by the thermal pretreatment, no significant differences were observed for diatoms. Indeed, both of them present a resistant cell wall, but their characteristics and composition differ. On the one hand, *Chlorella* sp. has mainly a carbohydrate-based cell wall, and carbohydrates solubilization can be boosted by the thermal pretreatment [30]. On the other hand, diatoms have a siliceous-based cell wall, which resists the effect of temperature.

In spite of this, both microalgae species were partially removed during the AD process according to digestate counting. While *Chlorella* showed around one logarithmic unit removal, a much lower removal efficiency was observed for diatoms, leading to a higher relative abundance of diatoms in the digestates. Comparing both *Chlorella* and diatom abundance in untreated and pretreated microalgae digesters, no significant differences were found. Even so, the pretreated microalgae digester showed a higher methane yield and VS removal. This may be because, although having same quantity of entire cells, those cells that were attacked by microorganisms were more degraded in the pretreated microalgae reactor.

### 2.3. Effect of the HRT on Microalgae Anaerobic Biodegradability

**Figure 5:**
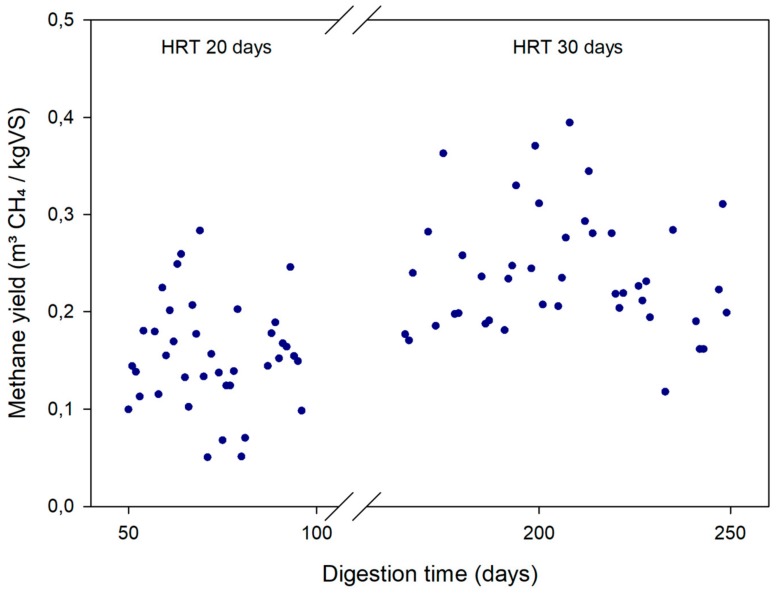
Daily methane yield of thermally pretreated microalgae for the two studied periods: Period I at an HRT of 20 days and Period II at an HRT of 30 days.

The effect of the HRT can be evaluated by comparing the results on pretreated microalgae AD obtained in both periods (at 20 and 30 days of HRT). When the HRT was increased to 30 days, the methane yield of pretreated microalgae increased by 50% (from 0.16 to 0.24 L CH_4_/g VS) compared to that obtained at 20 days of HRT (Table 3, Figure 5). Indeed, the VS removal was also higher with a HRT of 30 days (39.5%) as compared to 20 days (27.9%).

Although one expected benefit of applying a pretreatment is the kinetics improvement and thereby a reduction of the HRT [7], the methane yield increase reported in this study was still significant when he HRT was increased from 20 to 30 days. Thus, operating microalgae digesters at moderate HRTs seems appropriate, even if applying pretreatments. As discussed in the previous section, the thermal pretreatment weakened the microalgae cell wall but without completely lysing and releasing all intracellular material. Therefore, increasing the HRT enhanced the chance for microorganisms to access microalgae intracellular material through their weakened or damaged cell wall. These results are in agreement with previous studies. For instance, applying a thermal pretreatment to microalgae did not show any significant differences with a HRT of 15 days, but it increased the methane yield by 72% with a HRT of 20 days [8]. It has been suggested that the operation of digesters at high sludge retention times (SRT) promotes the presence of low growth-rate microorganisms and increases the hydrolytic potential of the system [31]. Comparing a thermophilic continuous stirred tank reactor working at 50 days of HRT (and SRT) with an anaerobic membrane bioreactor (AnMBR) with a SRT of 70 days, higher microbial diversity could be found in digesters working at higher HRT system [31].

## 3. Discussion

Results have shown how the co-digestion with primary sludge can substantially improve the microalgae mono-digestion by increasing the methane yield, decreasing the ammonia concentration, which may enable increasing the OLR, and improving the digestate dewaterability.

This study assessed different proportions of primary sludge and microalgae in batch tests and determined the best one in continuous lab-scale reactors. The truth is that in full-scale microalgal-based WWTPs, this proportion would change over the year. Indeed, the microalgal biomass production shows a strong seasonality [27] depending on the HRAPs operation conditions, influent characteristics, etc. [32]. These factors determine not only the amount but also the microalgae species in the system [27,33], and the microalgae species also affect the anaerobic digestion rate and extent, depending especially on the characteristics of the cell wall [27]. Overall, the implementation of anaerobic digesters in HRAP plants involves working with different proportions of microalgae and primary sludge and different microalgae species over the year. All these factors should be considered when it comes to sizing an AD plant integrated to a HRAP system. For instance, if the proportion of primary sludge is expected to be high, the biogas production is also expected to be high, and the operation of the digesters should be feasible at 20 days of HRT. However, if the proportion of microalgae is expected to be high, then it is necessary to assess the most appropriate strategy to follow (increasing the HRT and/or applying a pretreatment).

In this study, the thermal pretreatment increased the microalgae methane yield, but not as much as expected due to the presence of microalgae species with hardly degradable cell walls (i.e., *Stigioclonium* sp.). However, when the reactors were operated at a longer HRT (30 days), the methane yield of pretreated microalgae increased considerably (from 0.16 to 0.24 L CH_4_/g VS). When considering these alternatives, different issues should be addressed: firstly, the balance between the energy requirements in comparison the energy gain of the pretreatment step; secondly, the increase of volume, surface area and costs resulting from an increased HRT.

Consequently, an energy assessment was carried out by scaling-up the results of the lab-scale reactors during both experimental periods (I: co-digestion vs. pretreated microalgae mono-digestion at 20 days of HRT; II: pretreated vs. untreated microalgae at 30 days of HRT). Flow rates between 10–100 m^3^/day were considered (Table 4). The assessment compared the energy required to apply the pretreatment (if any) and anaerobic digestion (Ei) with the energy obtained through the biogas produced in each case (Eo). In this way, when the energy ratio (Eo/Ei) is higher than 1, there is an energy gain. As can be seen in Table 4, this value was higher than 1 in all scenarios, meaning that the energy balance was always positive. However, the best results were obtained with the co-digestion of microalgae and primary sludge (energy ratio between 3.5–4). This means that the energy produced with the co-digestion is at least 3.5-fold the energy consumed. Regarding the thermal pretreatment, this also showed an energy gain in all cases. However, the energy ratio increased from 1.1–1.2 to 1.5–1.7 by increasing the HRT from 20 to 30 days. When comparing the energy gain with untreated and pretreated microalgae at the same HRT of 30 days, the results are very similar (from 1.4–1.6 to 1.5–1.7). Bearing in mind the investment and operation costs of the pretreatment, this would not be worthwhile in terms of energy production, and would only become so if other benefits such as hygenisation were considered.

To sum up, the most suitable option to anaerobically digest microalgae from HRAPs would be the co-digestion with primary sludge at a 20-day HRT if the proportion of sludge was high, and at 30 days if the proportion of microalgae was high. The energy gain could be used to cover the energy demand of the WWTP, moving towards energy-neutral WWTPs [32]. Thus, integrating anaerobic co-digestion in HRAPs is a good strategy to transform resources from wastewater into valuable products. It enables resource recovery from wastewater, which is a prerequisite for the technological development of a cradle-to-cradle bio-based economy [34].

## 4. Materials and Methods

### 4.1. Substrates Caracteristics

The microalgal biomass (hereafter called microalgae) used in this study consisted of a microalgae-bacteria consortium grown in a pilot raceway pond (0.5 m^3^) that treated wastewater from a municipal sewer, as described by Passos et al. [27]. Microalgal biomass was harvested from secondary settlers and gravity thickened in laboratory Imhoff cones at 4 °C for 24 h. The pilot plant was located at the laboratory of the GEMMA research group (Barcelona, Spain).

Thickened primary sludge and digested sludge used as inoculum in BMP tests and continuous reactors came from a municipal WWTP near Barcelona. The inoculum was collected before the start-up of each assay, while primary sludge was periodically collected (every 3 weeks) and stored at 4 °C before use.

Thickened microalgae presented an average concentration of 3.7% TS and 2.7% VS, while primary sludge had an average concentration of 4.6% TS and 3.4% VS. In order to use the same OLR in all digesters, both substrates were diluted to achieve 2.5% VS.

### 4.2. Thermal Pretreatment

The thermal pretreatment of microalgae was carried out in glass bottles with a total volume of 250 mL and a liquid volume of 150 mL Bottles were placed in an incubator under continuous stirring at a constant temperature of 75 °C for 10 h. In semi-continuous experiments, microalgae were collected and pretreated once a week. Pretreated biomass was then stored at 4 °C before use.

### 4.3. Biochemical Methane Potential Tests

BMP tests were used to study the anaerobic biodegradability of co-digestion trials of primary sludge and microalgae, with and without thermal pretreatment. To this end, three proportion conditions were tested: (i) 25% of microalgae and 75% of primary sludge, (ii) 50% of microalgae and 50% of primary sludge and, (iii) 75% of microalgae and 25% of primary sludge on a VS basis. All conditions were conducted with untreated and pretreated microalgae.

The substrate to inoculum (S/I) ratio was 0.5 g COD/g VS, according to Arias et al. [35]. After adding the proper amount of both substrates and inoculum, serum bottles (160 mL) were filled with distilled water up to 100 mL, flushed with helium gas, sealed with butyl rubber stoppers and incubated at 35 °C until biogas production ceased. Accumulated biogas was measured with a manometer (GMH 3161 Greisinger, Regenstauf, Germany) and the methane content in biogas was periodically analyzed by gas chromatography. A blank treatment was used to quantify the amount of methane produced by the inoculum alone. Each co-digestion condition was performed in duplicate, whereas control trials (microalgae, pretreated microalgae and primary sludge) and blank were performed in triplicate.

### 4.4. Continuous Anaerobic Digestion

Microalgae anaerobic (co-)digestion was performed and monitored using two lab-scale reactors (2 L), with an effective volume of 1.5 L. Reactors were operated under mesophilic conditions (37 ± 1 °C) by implementing an electric heating cover (Selecta, Barcelona, Spain). Constant mixing was provided by a magnetic stirrer (Thermo Scientific, Waltham, MA, USA). Reactors were operated on a daily feeding basis, where the same volume was purged from and added to digesters using plastic syringes.

During the first experimental period, one of the digesters was fed with pretreated microalgae (i.e., control), while the second one was fed with pretreated microalgae (25% VS) and primary sludge (75% VS). Both reactors were operated at a HRT of 20 days and were considered to be under steady-state after 2.5 HRTs. Afterwards, the anaerobic digestion performance was further monitored during 2 complete HRTs (~6 weeks). During the second experimental period, the HRT was increased to 30 days. One reactor was still fed with pretreated microalgae, while the other one was fed with untreated microalgae (i.e., control). They were also considered to be under steady-state after 2.5 HRTs and anaerobic digestion performance was further monitored during 2 complete HRTs (~8.5 weeks). The total operation period of the digesters was 225 days.

Biogas production was measured by the water displacement method and the methane content in biogas was periodically analyzed by GC. The volume of biogas produced was expressed under standard temperature (0 °C) and pressure (1 atm) conditions (STP).

### 4.5. Microscopic Analysis

Microalgae were periodically identified over the semi-continuous reactors operation. The analysis was carried out with an optic microscope (Motic BA310E, Motic, Hong Kong, China), equipped with a camera (NiKon DS-Fi2, Nanjing, China) using the software NISElements Viewer (Prague, Czech Republic). Microalgae genus were identified from classical specific literature [36,37].

To prove the effect of the thermal pretreatment and AD on microalgae, four sampling campaigns were conducted. In each campaign the following samples were analyzed: (i) untreated microalgae; (ii) thermally pretreated microalgae; (iii) effluent (digestate) from untreated microalgae AD and (iv) effluent (digestate) from pretreated microalgae AD. From these samples, microalgae species were identified and two of the most abundant were quantified (*Chlorella* sp. and diatoms). For their quantification, each well homogenized sample was examined by bright and contrast phase microscopy using a Zeiss microscope Axioskop 40 (Goettingen, Germany). To quantify *Chlorella* sp. and diatoms, two subsamples of 20 µL, were counted at 400 magnification. In each subsample, 30 microscopic fields across the cover-slide were counted using coverslides of 20 mm side [38]. Previous to the cell counting, aggregated flocs of these unicellular species were broken down by means of an ultrasound technique [39].

### 4.6. Analitical Procedures

The TS and VS analysis was performed according to the standard methods [40]. The quantification of total COD concentration was performed according to the closed reflux colorimetric method outlined by the standard methods [40]. TKN was determined by titration after a mineralization step performed by a BUCHI 370-K distillator/titrator. The concentration of the ammonium nitrogen (N-NH_4_^+^) was measured according to the method by Solorzano [41]. pH was determined with a Crison Portable 506 pH-meter (Alella, Spain). Digestate dewaterability was evaluated by means of the capillary suction time (CST) test (Triton Electronics Ltd., Cambridge, UK). Volatile fatty acids (VFA) concentrations in continuous flow digesters were measured once a week by injecting 1 µL of each sample, once centrifuged (4200 rpm for 8 min) and filtered (0.2 µm), into an Agilent 7820A GC (Santa Clara, USA) after sulphuric acid and diisopropyl ether addition. The GC was equipped with an auto-sampler, flame ionization detector and a capillary column (DP-FFAB Agilent 30 m × 0.25 mm × 0.25 µm), and operated at injector and detector temperatures of 200 and 300 °C, respectively, with helium as carrier gas.

Biogas composition was determined by calculating the percentage of methane and carbon dioxide in the digesters headspace. Gases were measured by means of a GC (Thermo Finnigan, Austin, TX, USA) equipped with a thermal conductivity detector (TCD) (Hayesep packed column). The carrier gas was helium and injector/detector/oven temperatures of 150/250/35 °C, respectively. The methane content in biogas from BMP tests was measured each sampling day, while in continuous reactors it was quantified twice a week.

### 4.7. Statistics and Kinetic Data Analysis

The effect of independent variables during the continuous anaerobic (co-)digestion was evaluated via multi-factor analysis of variance (ANOVA) considering a 95% confidence level (α = 0.05) using the R Statistics Software. For the BMPs and the microscopic counting, mean values and standard deviations were considered.

To evaluate the kinetics of the process from BMP tests results, experimental data was adjusted to a first-order kinetic model (Equation (1)) by the least square method.

B = B_0_ {1 − exp[−k·t]} (1)

where B_0_ stands for the methane production potential (mL CH_4_/g VS), k is the first order kinetic rate constant (day^−1^), B is the accumulated methane production at time t (mL CH_4_/g VS) and t is time (day).

The error variance (*s*^2^) was estimated by the following equation (Equation (2)):

(2) $$s^{2}=\frac{\sum_{1}^{i}{\left(y_{i}-\hat{y_{i}}\right)}^{2}}{N-K}$$

where *y_i_* is the experimental value, *ŷ_i_* is the value estimated by the model, *N* is the number of samples and *K* is the number of model parameters.

### 4.8. Energy Assessment

The theoretical energy balance of full-scale reactors was estimated by up-scaling experimental data to medium-size WWTP with flow rates of 10-25-100 m^3^/day. Electricity and heat requirements for microalgae pretreatment and anaerobic digestion were calculated according to Passos and Ferrer [8].

Input heat was calculated as the energy required to heat influent biomass from ambient temperature (Ta) to digestion temperature (Td), according to Equation (3) The density (ρ) and specific heat (γ) of microalgae and primary sludge were assumed to be the same as those of water, 1000 kg/m^3^ and 4.18 kJ/(kg·°C), respectively. Heat losses through the reactor wall were considered and the heat transfer coefficient (k) was assumed to be 1 W/(m^2^·day). The reactor wall surface area was calculated from the reactor useful volume, considering a 2:1 diameter to height ratio, while the reactor bottom and top were not accounted for.

Ei,heat = ρ·Q·γ·(Td − Ta) + k·A·(Td − Ta)·86.4(3)

where Ei,heat is the input heat (kJ/day); ρ is the density (kg/m^3^); Q is the flow rate (m^3^/day); γ is the specific heat (kJ/(kg·°C)); Td is the anaerobic digestion temperature (37 °C); Ta is the ambient temperature (20 °C); k is the heat transfer coefficient (W/(m^2^·°C)); and A is the surface area of the reactor wall (m^2^).

When thermal pretreatment was involved, heat recovery was considered. Input heat was calculated as the energy required to heat influent biomass from Ta to pretreatment temperature (Tp), subtracted by the heat recovered when cooling down biomass from Tp to Td (Equation (4)). Heat would be recovered by means of a heat exchanger, with an efficiency φ of 85%.

Ei,heat = ρ·Q·γ·(Tp − Ta) − ρ·Q·γ·(Tp − Td) φ + k·A·(Td − Ta)·86.4(4)

where Ei,heat is the input heat (kJ/day); ρ is the density (kg/m^3^); Q is the flow rate (m^3^/day); γ is the specific heat (kJ/(kg·°C)); Td is the anaerobic digestion temperature (37 °C); Ta is the ambient temperature (20 °C); Tp is the pretreatment temperature (75 °C); φ is the heat recovery efficiency (85%); k is the heat transfer coefficient (W/(m^2^·°C)); and A is the surface area of the reactor wall (m^2^).

Furthermore, input electricity for anaerobic digestion was estimated as the energy required for biomass pumping and reactor mixing, which were assumed to be 1800 kJ/m^3^ and 300 kJ/(m^3^_reactor_ day), respectively (Equation (5)):

Ei,electricity = Q·θ + V·ω(5)

where Ei,electricity is the input electricity (kJ/day); Q is the flow rate (m^3^/day); θ is the electricity consumption for pumping (kJ/m^3^); V is the useful volume (m^3^); and ω is the electricity consumption for mixing (kJ/(m^3^_reactor_·day)).

The energy output of the process was calculated from the methane production rate of each reactor, according to Equation (6). The lower heating value of methane (ξ) was assumed to be 35,800 kJ/m^3^ CH_4_. An efficiency of 90% on energy conversion was considered (η).

Eo = P,CH_4_·ξ·V·η (6)

where Eo is the output energy (kJ/d); P,CH_4_ is the methane production rate (m^3^ CH_4_/(m^3^_reactor_·day)); ξ is the lower heating value of methane (kJ/m^3^ CH_4_); V is the useful volume (m^3^); and η is the energy conversion efficiency.

Finally, results were expressed as energy balance (ΔE) and energy ratio (Eo/Ei). The energy balance was calculated as the difference between the energy output and energy input (heat and electricity) (Equation (7)), while the energy ratio was calculated as the energy output over the energy input (heat and electricity) (Equation (8)).

ΔE = Eo − (Ei,heat + Ei,electricity)(7)

Eo/Ei = Eo/(Ei,heat + Ei,electricity)(8)

## Figures and Tables

**Table 1:** Experimental conditions during the mesophilic anaerobic digestion (AD) in lab-scale reactors. HRT: hydraulic retention time; VS: volatile solids.

	Period I	Period II
	(HRT = 20 Days)	(HRT = 30 Days)
Digester 1	25% VS pretreated ^1^ microalgae + 75% VS primary sludge	Untreated microalgae
Digester 2	Pretreated ^1^ microalgae	Pretreated ^1^ microalgae

^1^ 75 °C for 10 h.

**Table 2:** Ultimate methane yield (mean values ± standard deviation) and first-order kinetics constant (*k*) (error variance (s^2^) represented in brackets) obtained in the biochemical methane potential (BMP) test.

Trial	Methane Yield (mL CH_4_/g VS)		First-Order Kinetics (*k*) (Day^−1^)	
	Experimental Values ^1^	Calculated Values ^2^	Experimental Values ^1^	Calculated Values ^3^
Microalgae (M)	90 ± 2	-	0.07 (≤30)	-
75% M + 25% PS ^4^	133 ± 6	162	0.27 (≤74)	0.16 (70)
50% M + 50% PS ^4^	216 ± 1	234	0.28 (≤80)	0.20 (88)
25% M + 75% PS ^4^	291 ± 9	306	0.27 (≤108)	0.23 (113)
Pretreated Microalgae (Mp)	146 ± 6	-	0.16 (≤75)	-
75% Mp + 25% PS ^4^	183 ± 2	204	0.25 (≤85)	0.20 (72)
50% Mp + 50% PS ^4^	249 ± 17	262	0.28 (≤99)	0.22 (82)
25% Mp + 75% PS ^4^	339 ± 2	320	0.25 (≤150)	0.23 (107)
Primary Sludge (PS)	378 ± 4	-	0.24 (≤162)	-

^1^ Experimental data from BMP tests; ^2^ Theoretical values calculated as the sum of the ultimate methane yield of each substrate mono-digestion times their proportion in the trial; ^3^ Values obtained from the curves that represent the theoretical values calculated as the sum of the ultimate methane yield of each substrate mono-digestion times their proportion in the trial over time; ^4^ volatile solids basis.

**Table 3:** Biogas production, solids removal, influent (substrate) and effluent (digestate) characteristics from untreated or thermally pretreated microalgae AD and co-digestion with primary sludge in lab-scale reactors. Mean ± standard deviation. OLR: organic loading rate.

		Period I		Period II	
		Microalgae,p	Co-Digestion	Microalgae	Microalgae,p
**Operational Conditions**	HRT (days)	20	20	30	30
	OLR (g VS/L·day)	1.21 ± 0.06	1.17 ± 0.09	0.85 ± 0.01	0.81 ± 0.02
**Biogas Production**	Methane production rate (L CH_4_/L·day)	0.20 ± 0.05	0.53 ± 0.29 ^a^	0.12 ± 0.08	0.19 ± 0.07 ^b^
	Methane yield (L CH_4_/g VS)	0.16 ± 0.05	0.46 ± 0.27 ^a^	0.14 ± 0.07	0.24 ± 0.07 ^b^
	Methane content in biogas (% CH_4_)	66.2 ± 2.62	71.7 ± 0.9 ^a^	67.6 ± 1.6	69.5 ± 1.7
**Removal Efficiency**	TS removal (%)	16.6 ± 4.1	19.0 ± 1.7 ^a^	18.6 ± 1.7	26.2 ± 3.7 ^b^
	VS removal (%)	27.9 ± 1.9	34.3 ± 2.4 ^a^	36.2 ± 2.5	39.5 ± 3.7 ^b^
**Influent Characteristics**	TS [% (*w*/*w*)]	3.87 ± 0.28	4.13 ± 0.29	3.63 ± 0.48	3.42 ± 0.28
	VS [% (*w*/*w*)]	2.47 ± 0.17	2.38 ± 0.15	2.42 ± 0.14	2.37 ± 0.10
	VS/TS (%)	64 ± 3 ^a^	58 ± 3	56 ± 2	55 ± 2
	COD (g O_2_/L)	42.0 ± 6.7	42.9 ± 7.7	26.6 ± 1.6	25.2 ± 1.8
	TKN (g/L)	n.a.	n.a.	2.4 ± 0.1	2.3 ± 0.1
	N-NH_4_ (g/L)	0.16 ± 0.07	0.13 ± 0.06	0.06 ± 0.01	0.26 ± 0.06 ^b^
**Effluent Characteristics**	pH	7.55 ± 0.15 ^a^	7.30 ± 0.08	7.35 ± 0.11	7.55 ± 0.08 ^b^
	TS [% (*w*/*w*)]	3.49 ± 0.34	3.53 ± 0.18	2.87 ± 0.16	2.67 ± 0.27
	VS [% (*w*/*w*)]	1.77 ± 0.09 ^a^	1.62 ± 0.11	1.58 ± 0.06	1.45 ± 0.11
	VS/TS (%)	51 ± 3 ^a^	46 ± 2	56 ± 2	55 ± 2
	COD (g/L)	30.9 ± 2.1	29.0 ± 3.0	26.6 ± 1.6	25.2 ± 2.1
	N-NH_4_ (g/L)	1.1 ± 0.2 ^a^	0.6 ± 0.1	0.7 ± 0.1	0.8 ± 0.1
	VFA (mg COD/L)	124 (<756 ^1^)	44 (<757 ^1^)	0 (<0 ^1^)	130 (<596 ^1^)
	CST (s)	982 ± 61 ^a^	290 ± 11	795 ± 71	919 ± 21 ^b^

^1^ Maximum value achieved. p = pretreated; TKN = total Kjeldahl nitrogen; VFA = volatile fatty acids; CST = capillarity suction time. ^a,b^ Stand for significantly higher values between paired columns (“a” for period I and “b” for period II) (α = 0.05).

**Table 4:** Results of the energy assessment for the co-digestion and pretreated microalgae mono-digestion at 20 days of HRT, and for the untreated and pretreated microalgae mono-digestion at 30 days of HRT, with different flow rates (Q = 10, 25 and 100 m^3^/day). Ei (i.e., energy input) and Eo (i.e., energy output).

	Period I						Period II					
	Microalgae,p			Co-Digestion			Microalgae			Microalgae,p		
Q (m^3^/day)	10	25	100	10	25	100	10	25	100	10	25	100
Ei (GJ/day)	1.15	2.75	10.46	0.96	2.28	8.58	0.99	2.31	8.53	1.24	2.93	11.04
Eo (GJ/day)	1.29	3.22	12.89	3.42	8.54	34.15	1.35	3.38	13.53	1.84	4.59	18.37
∆E = Eo − Ei (GJ/day)	0.14	0.47	2.43	2.45	6.26	25.27	0.36	1.08	5.00	0.60	1.66	7.32
Eo/Ei (-)	1.1	1.2	1.2	3.5	3.7	4.0	1.4	1.5	1.6	1.5	1.6	1.7
